# Extracellular‐Vesicle mediated pyroptosis leads to gut‐brain axis after stroke in a mouse model of Alzheimer’s Disease

**DOI:** 10.1002/alz.089237

**Published:** 2025-01-03

**Authors:** Nadine A Kerr, Alfredo Fernandez Higueras, Juliana Sanchez‐Molano, Winston Walters, Juan Pablo de Rivero Vaccari, Robert W Keane, Helen M Bramlett, W Dalton Dietrich

**Affiliations:** ^1^ The Miami Project to Cure Paralysis, Miami, FL USA; ^2^ University of Miami, Miami, FL USA; ^3^ Bruce W. Carter Veterns Affairs Medical Center, Miami, FL USA

## Abstract

**Background:**

Stroke and AD patients with gut complications often present worsened neurological outcomes. The goal of this study was to examine the role of extracellular vesicle (EV)‐mediated pyroptosis in the bi‐directional gut‐brain axis after photothrombotic stroke (PTS) in aged 3xTg mice and wildtype (WT) controls.

**Method:**

Twelve‐month 3xTg and WT male and female mice underwent PTS using a YAG laser. Lesion volume analysis was performed at 1‐month post‐PTS. At 24 hours after PTS, intestinal and cortical tissue was collected for Western Blot analysis for the following inflammasome proteins: caspase‐1, apoptosis‐associated speck‐like protein containing a caspase recruitment domain (ASC), interleukin‐1b, and Gasdermin‐D (GSDMD). Additionally, brain and intestines were sectioned for immunohistochemical analysis of GSDMD and Amyloid‐Beta (Ab). In order to show changes in gut function, gut permeability was measured 72 hours post PTS using a FITC‐dextran assay. Moreover, we performed an adoptive transfer in which stool‐derived EVs collected from 3xTg‐ PTS and WT‐PTS mice were injected into naïve 3xTg and WT mice. Cortical lysates were examined for expression of inflammasome proteins, GSDMD, and Ab using Western Blot analysis.

**Results:**

3xTg mice presented increased infarct volume compared to WT‐PTS mice one month after PTS. Based on Western blot analysis, we found that inflammasome proteins, GSDMD, and Ab were significantly increased in both intestinal and cortical tissue in 3xTg‐ PTS mice compared to WT‐ PTS mice at 24 hours post‐stroke. Results from the FITC‐Dextran assay showed that gut permeability was significantly increased in the aged 3xTg‐PTS mice compared to WT‐ PTS mice 72 hours post‐PTS. Additionally, our immunohistochemical analysis in both WT and 3xTg PTS mice presented evidence of activated/ameboid microglial morphology as well as the presence of GSDMD and Ab in the brain and intestines 72 hours after stroke. Lastly, we demonstrated increased levels of inflammasome proteins, GSDMD, and Ab after adoptive transfer.

**Conclusion:**

Taken together these results indicate an important role for EV signaling and pyroptosis in disruption of the bidirectional gut‐brain axis after stroke in aged 3xTg mice in an animal model of stroke.

